# Why are some people more fit than others? Correlates and determinants of cardiorespiratory fitness in adults: protocol for a systematic review

**DOI:** 10.1186/s13643-017-0497-4

**Published:** 2017-05-18

**Authors:** Nita Perumal, Gert B.M. Mensink, Thomas Keil, Jonas David Finger

**Affiliations:** 1 Department of Epidemiology and Health Monitoring, Robert Koch Institute, General-Pape Str. 62-66, 12101 Berlin, Germany; 2 Institute for Social Medicine, Epidemiology and Health Economics, Charité University Medical Center Berlin, Luisenstr. 57, 10117 Berlin, Germany

**Keywords:** Cardiorespiratory fitness, Aerobic fitness, Correlates, Determinants, Risk factors, Systematic review

## Abstract

**Background:**

Cardiorespiratory fitness (CRF) is a physical condition that is now well established as a predictor of numerous adverse health outcomes, independent of physical activity levels. In order to be able to improve CRF at the population level and to develop effective interventions and public health programmes, it is important to understand why some people are more fit than others. Therefore, the primary aim of the systematic review described in this protocol is to examine individual and interpersonal factors that are correlated with or determine CRF among adults.

**Methods:**

The review will focus on quantitative studies that investigate any personal and interpersonal correlates and/or determinants of objectively measured CRF among the general, non-symptomatic, non-institutionalized adult population (aged 18–65 years) worldwide. The databases MEDLINE, Embase, and Cochrane Library will be searched to identify all relevant published journal articles, and Google Scholar and Scopus will be searched for grey literature. Studies where CRF is not an outcome variable and experimental studies where participants specifically receive a fitness intervention that increases CRF will be excluded. For each study, data extracted will include, among other variables, study characteristics, methodology for selecting participants into the study as well as the participants’ demographic characteristics, types of correlates and determinants of CRF investigated and their measurement methods, the objective measure of CRF used and its measurement method and validity, and the main reported results on the association between the correlates or determinants and CRF. In addition, observational studies will be assessed for methodological quality and risk of bias using a customized version of the Quality Assessment Tool for Observational Cohort and Cross-Sectional Studies by the National Heart, Lung, and Blood Institute. Experimental studies will be assessed using the 27-item Downs and Black “Checklist for Measuring Study Quality”. The final results will be presented as a narrative synthesis of the main findings of all included studies.

**Discussion:**

By consolidating and synthesizing the current research on possible individual and interpersonal correlates and determinants of CRF among adults worldwide, we aim to aid future public health actions, as well as identify gaps in our full understanding of what influences CRF.

**Systematic review registration:**

PROSPERO CRD42017055456.

**Electronic supplementary material:**

The online version of this article (doi:10.1186/s13643-017-0497-4) contains supplementary material, which is available to authorized users.

## Background

Physical activity (PA) has long been a focus of public health and epidemiological research [1], and reducing levels of physical inactivity is a central goal in the global strategy to reduce and prevent non-communicable diseases [2]. Our current comprehensive understanding of PA encompasses not only its wide-ranging health benefits [3–7] but also which factors are associated with or influence its varying levels among different individuals [8–10]. Physical fitness, specifically cardiorespiratory fitness (CRF), refers to another dimension of our physical health and occurs partially, but not only [11], as result of PA [12–15]. CRF is now equally well established as a predictor, independent of PA levels, of numerous health outcomes, such as adverse cardiovascular events and all-cause mortality [16], cancer mortality [17], metabolic syndrome [18], and depressive symptoms [19]. This can be explained by the fact that CRF is often an objectively measured physical condition that is defined as “the ability of the circulatory and respiratory systems to supply oxygen during sustained physical activity” ([20], p. 52), while PA is often a self-reported behaviour that is defined as “any bodily movement produced by skeletal muscles that requires energy expenditure” ([20], p. 53). The two signify different stages within the causal pathway towards various disease states. Currently, there is a clear consensus among the research community that CRF is as important as PA [21–23], if not even more so [24], in determining future adverse health outcomes. The American Heart Association has even recommended the establishment of a national registry in the USA in order to better track CRF among the population and facilitate the availability of more comprehensive data for research [25].

**Fig. 1:**
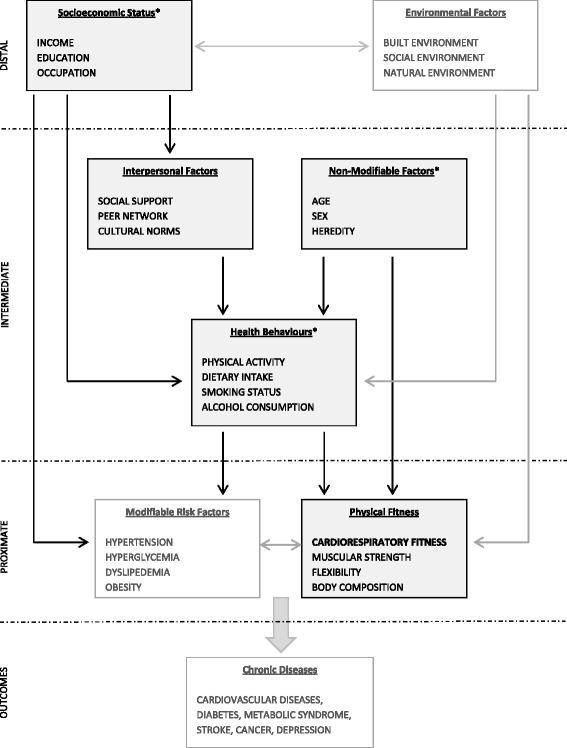
Conceptual framework for determinants of chronic disease outcomes, with focus on cardiorespiratory fitness. *Asterisk* categorized as “individual factors”

However, in order to be able to improve CRF at the population level and to develop effective interventions and public health and clinical programmes, not only do we need to know the prevalence of low versus high CRF in the population but also why some people are more fit than others. Just as PA and CRF independently predict adverse health outcomes, the factors underlying low CRF may be distinct from those underlying insufficient PA or may have differing relationship patterns. Hence, investigating the factors that are specifically associated with or influence CRF is crucial. Researchers have begun to focus more and more on examining such relationships, and there has been a steady accumulation of studies linking a number of factors to CRF, even after adjustment for PA. Examples of some factors are heritability [26], age and adiposity [27], early childhood growth [28], smoking [29], alcohol consumption [30], parental affluence [31], physically demanding occupational work [32], and residential built environment [33]. Figure 1 proposes a broad conceptual framework of factors that influence chronic disease outcomes, including CRF. Using the methodology outlined by Victora et al. [34], the framework is organized in a hierarchical manner and is adapted from three ecological models: the Dahlgren-Whitehead model of determinants of health [35], the World Health Organization model of causes of chronic diseases [36], and the Lancet Physical Activity Series Working Group model of determinants of physical activity [8]. The conceptual model groups socioeconomic factors and environmental factors as distal factors that influence chronic disease outcomes; interpersonal factors, non-modifiable biological factors, and health behaviours as intermediate; and modifiable risk factors and physical fitness as proximate in the hierarchy. The proposed framework is not exhaustive and has been simplified to represent the position of CRF in the pathway and highlight its proximity to the onset of chronic diseases and its relationships with factors that are intermediate and distal in the hierarchy.

Whereas the proposed conceptual framework has a broad scope, the scope of this systematic review is narrower and focuses on the socioeconomic factors, non-modifiable biological factors, and health behaviours that determine CRF (together referred to as “individual factors”), as well as the interpersonal factors that determine CRF. In particular, the aim of this systematic review is to examine the individual and interpersonal factors that are associated with CRF (hereafter referred to as “correlates”) or influence CRF (hereafter referred to as “determinants”) among the general adult population worldwide.

## Methods

### Design

This systematic review protocol will follow the guidelines outlined in the Preferred Reporting Items for Systematic Review and Meta-Analysis Protocols (PRISMA-P) 2015 Statement. Additional file 1 contains the completed PRISMA-P checklist.

### Types of studies

Our review will include quantitative observational studies (cohort studies, case-control studies, and cross-sectional surveys) and experimental studies (randomized controlled trials, non-randomized controlled trials, and quasi-experimental studies) that investigate correlates and/or determinants of CRF in the general adult population. Studies that aim to increase participants’ CRF with exercise training interventions will be not be included in this review. Such studies are outside the scope of our research question as they focus on the role of specific types of exercises or other physical activity interventions, often with set durations and intensities, in improving CRF over a pre-specified period of time.

### Types of participants

Our review will include studies that report on correlates and/or determinants of CRF among adults aged 18–65 years of age in the general population (i.e. non-symptomatic, non-institutionalized populations) and will exclude results for children and adolescents, as well as the elderly. We will further exclude studies where the participants are not representative of the general population and are instead selected from specific population subgroups from which the results cannot be generalized. This includes, for example, adults with disease conditions such as coronary heart disease, chronic pulmonary obstructive disease, or arthritis, or occupational groups requiring high levels of physical fitness, such as military personnel, professional athletes, or firefighters. Studies that restrict their study participants to one sex only will be included as long as the participants are representative of the general population for their sex.

### Types of exposures

Since this review aims to elucidate all potential individual and interpersonal factors that are correlated with or influence CRF, all research studies that investigate the relationship between CRF and any one or more individual factors (non-modifiable biological risk factors, behavioural risk factors, and socioeconomic risk factors) and/or interpersonal factors will be included. Within the so-called individual factors, specific examples of non-modifiable biological risk factors are age and race; specific examples of behavioural risk factors are dietary intake, physical activity, and smoking status; and specific examples of socioeconomic risk factors are income and completed education. Specific examples of interpersonal factors are spousal social support, single-parent status, and social interaction.

### Types of outcomes

Our review will focus on objectively assessed measures of CRF as the main outcome. By “objectively assessed”, we mean CRF that is measured through maximal, or estimated through submaximal, treadmill or cycle ergometer tests. Maximal ergometer tests are the gold standard for the measurement of CRF [37, 38], but due to the vigorous exertion required, they carry the risk of causing adverse cardiac outcomes [39] and require the presence of a trained physician and full set-up of safety and emergency equipment [37]. Therefore, submaximal tests are used frequently in studies, especially in larger, population-based studies [40, 41], to measure CRF instead because they are safer as well as more cost-effective and time-effective, while still providing an objective and satisfactory assessment of CRF [42, 43].

### Search strategy

We will conduct our search in the following literature databases for articles published in journals:

- MEDLINE, 1946–present (indexed citations via PubMed.com)
- EMBASE (via Embase.com)
- Cochrane Library (via Wiley Online Library)

In addition, we will conduct a grey literature search in the following databases:

- Scopus
- Google Scholar

A draft search strategy was first developed for the MEDLINE database using a combination of the most relevant and appropriate Medical Subject Headings (MeSH) and title/abstract text keywords for CRF measures and tests (outcome), as well as general categories of correlates and determinants (exposure). A preliminary screening of the first 500 citation results was used to inform the development of an updated and improved search strategy that will be used for the final MEDLINE search and will also serve as a guide for the development of the search strategy for the other databases of interest. Additional file 2 contains the final MEDLINE search strategy and rationale.

No date, language, article type, text availability, or species restrictions or filters will be applied for any of the searches. Search results from the different databases will be imported into the reference management software Endnote X7 (Thomson Reuters, USA) and de-duplicated as necessary. Reference lists of the final publications selected for extraction will be searched for any additional relevant references (published articles as well as grey literature) not found through the database searches.

### Selection of studies

The primary reviewer (NP) and the second reviewer (JF) will independently conduct title and abstract screening on all publications obtained through the literature search to identify those that are relevant to the research question and meet the inclusion and exclusion criteria (Table 1).

**Table 1:** Inclusion and exclusion criteria for screening of search results

Inclusion criteria	Exclusion criteria
• Primary results report on association (or lack of association) between one or more personal and/or interpersonal factor(s) and CRF • CRF measured through maximal or submaximal ergometry test • Results reported for adults (18–65 years) in the general population	• CRF considered as an exposure variable (rather than as an outcome variable) in the study design and analysis • Study conducted in a highly selected population. Examples are: − Patients with specific disorders or medical conditions (e.g. coronary artery disease, heart failure, schizophrenia, chronic obstructive pulmonary disease) − Individuals categorized as obese − Military personnel − Professional athletes • Randomized controlled trial or another type of quasi-experimental design where participants receive a fitness or exercise training intervention that increases CRF • Qualitative study design (focus groups, open-ended interviews, and thematic reviews) • Editorials, letters to the editor, or commentaries

Publications that pass the initial screening will then undergo full-text review by the two reviewers independently and studies that meet all inclusion and exclusion criteria will be selected for data extraction. At all stages, disagreements between the first and second reviewers regarding inclusion or exclusion of studies will be resolved via discussion. The Endnote software will be utilized to sort and chronicle all included and excluded articles during the full-text screening process and a custom field will be created within the Endnote citations to document the reason for each article’s exclusion. The number of publications identified, included, and excluded at each screening stage, as well as the reason for their exclusion, will be outlined in a PRISMA flow diagram.

### Data extraction

Data will be independently entered into a pre-designed extraction form in Microsoft Excel by the two reviewers (NP and JF); the form will first be pilot-tested on five studies and improvements and updates will be implemented before data from the remaining selected studies are extracted. Any ambiguity or uncertainty in study data being extracted will be resolved via discussion with the second reviewer (JF) and, if necessary, a third reviewer (GM). The following information will be extracted from all studies:

1. Study characteristics: authors, title, study design, country, year of publication, and time period
2. Methods: research question, selection of study participants (sampling method, inclusion/exclusion criteria), sample size, and response rate/loss to follow-up
3. Population characteristics: target population under study; inclusion/exclusion criteria; age group; sex breakdown; and reported clinical, socioeconomic, behavioural, occupational, and environmental characteristics
4. Exposure variables: types of correlates or determinants of CRF studied, the primary correlates or determinants measured, confounders measured/adjusted for, mediation or interaction variables adjusted for, and measurement methods or instruments for each variable
5. Outcome variables: CRF measure used, measurement method or instrument, validity of the measurement method or instrument, and criteria for stopping assessment of CRF (where applicable)
6. Main results: method of statistical analysis; reported associations between the correlates or determinants and CRF, such as effect measures (odds ratios, risk ratios, or regression coefficients, with corresponding confidence intervals) and correlation coefficients (Pearson correlation or Spearman rank correlation); main results in total sample and by sex (where reported); and crude and adjusted associations (where reported)
7. Major study limitations noted by authors
8. Conflict of interest and funding

### Quality assessment

A customized version of the *Quality Assessment Tool for Observational Cohort and Cross-Sectional Studies* [44] by the National Heart, Lung, and Blood Institute at the National Institutes of Health, USA, will be utilized to assess the risk of bias and methodological quality for all selected observational studies. The first and second reviewers (NP and JF) will independently conduct the quality assessment for each study in order to ensure an objective evaluation process. The studies will be assessed based on questions addressing the following major criteria:

- Risk of selection bias:
  - Participant recruitment using probability-based sampling strategy and a national sampling frame
  - Appropriate inclusion/exclusion criteria and its uniform application
  - Adequate response rate, low loss to follow-up
- Precision:
  - Sample size justification
- Risk of information bias:
  - Measurement of exposure and outcome using valid and reliable instruments and a standardized methodology
- Adequate assessment of association between exposure and outcome:
  - Measurement of exposure prior to measurement (occurrence) of outcome
  - Investigation of dose-response relationship
  - Adjustment for key confounders in statistical analysis (e.g. physical activity, age, sex)
  - Sensitivity analysis
- Risk of investigator bias:
  - Source of funding
  - Statement regarding conflicts of interest

Additional file 3 provides the detailed questions for quality assessment. Studies will be given a cumulative quality rating of low, medium, or high based on responses to the questions. Any disagreements between the first and second reviewers will be resolved via discussion between all three reviewers (NP, JF, and GM) until an agreement is reached. While studies of all qualities will be included in the review, the limitations of studies rated low will be discussed and a sensitivity analysis will be conducted to assess their impact on any pooled analyses.

The risk of bias in all experimental studies included in the review will be assessed using the Downs and Black “Checklist for Measuring Study Quality” [45], which is a 27-item numerical checklist designed to assess the quality of both randomized and non-randomized studies (including quasi-experimental studies). The checklist has undergone pilot-testing and evaluation for both validity and reliability and has been given a methodological rating of “strong” [46]. The checklist consists of the following main dimensions:

- Reporting — assessment of the overall study quality
- External validity — assessment of whether the study results are generalizable
- Internal validity (measurement bias) — assessment of bias introduced when applying intervention and/or measuring outcome
- Internal validity (confounding and selection bias) — assessment of bias due to study sample selection and/or confounding
- Power — assessment of whether study results could be obtained by chance

As in the case of observational studies, the first and second reviewers (NP and JF) will independently conduct the quality assessment for each study. Each study will receive a total numerical score out of a maximum of 30 points and the inter-rater agreement will be calculated using the *kappa* statistic. For studies with a kappa statistic of <0.20, which is categorized as “poor agreement”, the reviewers will resolve disagreements via discussion. Where the first two reviewers cannot reach an agreement, the third reviewer (GM) will facilitate a resolution. Any studies that receive a total score of less than 15 will be specified and a sensitivity analysis would once again be performed in order to assess their impact on pooled analyses, if such analyses are conducted.

### Data synthesis

We plan to first conduct a narrative synthesis of the data from all included studies and provide a summary table that specifies, for each study, the major study design characteristics, participant attributes, investigated exposure and outcome variables, and main results and associations. We will synthesize our results separately for men and women due to the well-documented differences in cardiorespiratory fitness among the two sexes [47, 48]. If sufficient data are available from the included studies, we further plan to present our narrative results stratified by different exposure categories (e.g. age groups, smoking status, socioeconomic status); by study design (e.g. cross-sectional surveys or cohort studies); and by region or country of study. We will qualitatively assess the studies for heterogeneity and expect to find a fair number of differences, especially when comparing study populations, methodological designs, and exposures of interest. Therefore, a meta-analysis will only be conducted if more than two included studies are homogenous with regard to exposure, population characteristics, and study design and their reported results are appropriate for pooling. If data are pooled, inverse variance weighting will be applied for the pooling method in order to account for the differences in standard errors between studies, with the studies with larger sample sizes and, thus, lower standard errors, receiving a greater weight in the pooled results. Heterogeneity between the studies will be assessed using the *I* ^2^ statistic and forest plots. Finally, publication bias will be estimated using a funnel plot.

## Discussion

With this review, we aim to consolidate and synthesize the current knowledge and research on all individual and interpersonal correlates and determinants of CRF at the population level. Our review will treat CRF as an objective indicator of physical health that is distinct from PA levels, with potentially distinct upstream factors that influence it. Following the completion of this review, we plan to utilize our findings in future research, where we will investigate, using primary data from a national health examination survey of German adults, the association between CRF and factors identified through the systematic review.

Our proposed systematic review protocol has a number of strengths. These strengths are the important and relevant topic; a well-designed conceptual model that forms the theoretical basis for the review; a comprehensive and broad search strategy, where the systematic reviewers carefully apply most of the inclusion/exclusion criteria during the screening stage, rather than through a narrow and automated search strategy; independent screening and extraction by two reviewers; and, finally, a customized and rigorous quality assessment criteria. Our review also has certain weaknesses. First is the potential for publication bias due to the lack of publication of negative results. This is a real and unavoidable issue in the field of epidemiology and public health and, although we plan to assess for the presence and degree of this bias via funnel plots, it is difficult to adjust for it; thus, our findings could be somewhat biased. Second, since a large majority of individual and interpersonal correlates and determinants of relevance in this review cannot be ethically manipulated in an experimental study, our review will mostly contain results from observational studies, making the inference of causal relationships difficult.

To our knowledge, our review will be the first of its kind to address this topic, especially while maintaining a very broad outlook and investigating all possible individual and interpersonal correlates and determinants of CRF. The final purpose of our review is not only to identify which factors are strongly or consistently associated with or influence CRF, but also to guide future research on CRF by identifying those areas where knowledge gaps exist in our full understanding of what can determine CRF in the general adult population.

## Additional files

Additional file 1: PRISMA-P 2015 checklist.

Additional file 2: MEDLINE search strategy.

Additional file 3: Quality assessment criteria.

## Abbreviations

CRF: Cardiorespiratory fitness
MeSH: Medical Subject Heading
PA: Physical activity
PRISMA-P: Preferred Reporting Items for Systematic Review and Meta-Analysis Protocols
