# Supplementary material for: Why are some people more fit than others? Correlates and determinants of cardiorespiratory fitness in adults: protocol for a systematic review
Source: Syst Rev. 2017 May 18;6:102. doi: 10.1186/s13643-017-0497-4 (PMC5437494; doi:10.1186/s13643-017-0497-4)
Supplement: Supplementary file 2 — MEDLINE search strategy. [file 13643_2017_497_MOESM2_ESM.docx]

**MEDLINE articles search strategy via PubMed.com**

**Outcome terms [Title/Abstract text terms]:**

- “cardiorespiratory fitness”
- “cardiovascular fitness”
- “physical fitness”
- “aerobic fitness”
- “exercise capacity”
- “VO2max”
- “VO2peak”

**Publication topic [major MeSH topic]**

- physical fitness
- physical fitness/physiology
- exercise
- exercise/physiology

**Exposure terms [all MeSH terms]:**

- “socioeconomic factors”
  - Encompasses occupational factors as well and is better than using the separate MeSH term “occupational exposure”, which is related to hazardous occupational conditions and gives results that are not relevant to our topic
- “risk factors”
- “health behavior”
- “residence characteristics”

**Study design [MeSH terms]:**

- “epidemiologic studies”
- “surveys and questionnaires”
- “controlled clinical trials”

**Population [MeSH terms]:**

- “adult”

**To be excluded - Most frequent disease conditions in preliminary search results [Title/Abstract text terms]**

- cardiac artery disease
- asthma
- arthritis
- chronic pulmonary obstructive disease, COPD
- heart failure
- stroke
- fibromyalgia

**Search string structure**

[Fitness AND (Exposure OR Study Design) AND Population] NOT (Disease Conditions)

| **Query** | **Items found** |
| --- | --- |
| **((**cardiorespiratory fitness**[tiab]** OR cardiovascular fitness**[tiab]** OR physical fitness**[tiab]** OR aerobic fitness**[tiab]** OR exercise capacity[ti] OR vo2max[ti] OR vo2peak[ti]**)** AND **(**physical fitness[majr] OR physical fitness/physiology[majr] OR exercise[majr] OR exercise/physiology[majr]**)** AND **((**socioeconomic factors[mesh] OR risk factors[mesh] OR health behavior[mesh] OR residence characteristics[mesh]**)** OR **(**epidemiologic studies[mesh] OR surveys and questionnaires[mesh] OR controlled clinical trials[mesh]**))** AND adult[mesh]**)** NOT **(**cardiac[tiab] OR asthma[tiab] OR arthritis[tiab] OR chronic pulmonary obstructive disease[tiab] OR copd[tiab] OR heart failure[tiab] OR stroke[tiab] OR fibromyalgia[tiab]**)** | 1887 |

Note: Search updated on 26 January, 2017.
