# Supplementary material for: Why are some people more fit than others? Correlates and determinants of cardiorespiratory fitness in adults: protocol for a systematic review
Source: Syst Rev. 2017 May 18;6:102. doi: 10.1186/s13643-017-0497-4 (PMC5437494; doi:10.1186/s13643-017-0497-4)
Supplement: Supplementary file 3 — Quality assessment criteria. [file 13643_2017_497_MOESM3_ESM.docx]

**Quality Assessment for Systematic Review**

The following questions closely follow the *Quality Assessment Tool for Observational Cohort and Cross-Sectional Studies* (retrieved 13 April 2016) by the National Heart, Lung, and Blood Institute at the National Institutes of Health, USA.

Link: [**http://www.nhlbi.nih.gov/health-pro/guidelines/in-develop/cardiovascular-risk-reduction/tools/cohort**](http://www.nhlbi.nih.gov/health-pro/guidelines/in-develop/cardiovascular-risk-reduction/tools/cohort)

**Note 1**

Questions in **blue** were added by the reviewers after discussion. The last two questions regarding funding source and conflict of interest, in particular, were based on the *STROBE Statement - Checklist of items that should be included in reports of observational studies* (retrieved 13 April 2016)

Link: <http://www.strobe-statement.org/index.php?id=available-checklists>

**Note 2**

Exposure variables = correlate(s) of cardiorespiratory fitness and potential confounders

Outcome variable = objective measure of cardiorespiratory fitness

1. Was the research question clearly stated?
   1. Were the correlates of cardiorespiratory fitness being investigated clearly stated?
   2. Was the outcome of cardiorespiratory fitness clearly stated?
2. Was the study population clearly defined?
3. How were the study participants selected into the study?
   1. Were the participants sampled using a probability-based sampling strategy?
   2. Was the sampling frame at a national level?
   3. If participants were selected from clusters, were the number of clusters ≥ 50?
   4. Were the inclusion and exclusion criteria for being in the study pre-specified and applied uniformly to all participants?
4. What was the sample size?
   1. Was a sample size justification or power description provided?
5. What was the response rate?
   1. What was the response rate of eligible participants >50%?
6. What was the loss to follow-up in cohort studies?
   1. Was loss to follow-up after baseline 20% or less?
7. Were the exposure variables clearly defined, valid, reliable, and implemented consistently across all study participants?
   1. Where applicable, were the correlate(s) of cardiorespiratory fitness and potential confounders objectively measured through validated instruments?
   2. Were the majority of exposure variables self-reported?
8. Was the outcome variable clearly defined, valid, reliable, and implemented consistently across all study participants?
   1. Was cardiorespiratory fitness measured using an objective, reliable, and validated methodology?
   2. Was cardiorespiratory fitness measured consistently for all participants?
9. Were the exposure variables measured prior to the outcome variables being measured?
10. For exposures that can vary in amount or level, did the study examine different levels of the exposure as related to the outcome (i.e. dose-response)?
11. Were key potential confounding variables measured and adjusted for statistically to account for their impact on the relationship between the exposure and outcome variables?
12. Did the study investigate interaction between exposure variables?
13. Was any sensitivity analysis conducted?
14. Did authors report their funding source and/or make a statement regarding the presence or absence of conflicts of interest?

**Response categories:**

- Yes
- No
- CD (cannot determine)
- NA (not applicable)
- NR (not reported)
- Number values (for Questions 4-6)
